# A comprehensive calibration of integrated magnetron sputtering and plasma enhanced chemical vapor deposition for rare-earth doped thin films

**DOI:** 10.1557/s43578-023-01207-2

**Published:** 2023-11-07

**Authors:** Zahra Khatami, Lukas Wolz, Jacek Wojcik, Peter Mascher

**Affiliations:** 1 Department of Electrical and Computer Engineering, University of New Brunswick, Fredericton, NB E3B 5A3 Canada; 2 Department of Engineering Physics and Centre for Emerging Device Technologies, McMaster University, Hamilton, ON L8S 4L7 Canada; 3 University of Tübingen, Geschwister-Scholl-Platz, 72074 Tübingen, Germany

**Keywords:** Sputtering, Plasma-enhanced CVD (PECVD) (deposition), Rare-earths, Thin films, Silicon oxide, Terbium

## Abstract

A new integrated deposition system taking advantage of magnetron sputtering and electron cyclotron-plasma enhanced chemical vapour deposition (IMS ECR-PECVD) is presented that mitigates the drawbacks of each fabrication system. This tailor-made system provides users with highly homogeneous and pure thin films with less undesired hydrogen and well-controlled rare-earth concentration compared to existing methods of rare-earth doping, such as metalorganic powders, sputtering, and ion implantation. We established the first comprehensive report on the deposition parameters of argon flow and sputtering power to achieve desired rare-earth concentrations in a wide composition range of terbium (Tb) doped-silicon oxide (Tb:SiO_x_) matrices including silicon-rich (*x* < 2), oxygen-rich (*x* > 2), and stoichiometric silicon oxide (*x* = 2). The deposition parameters to fabricate crystalline structure (Tb_2_Si_2_O_7_) in oxygen-rich samples are reported where Tb ions are optically active. IMS ECR-PECVD pushes the solubility limit of the rare-earth dopant in silicon films to 17 at.% for the desired future nanophotonic devices.

**Graphical Abstract:**

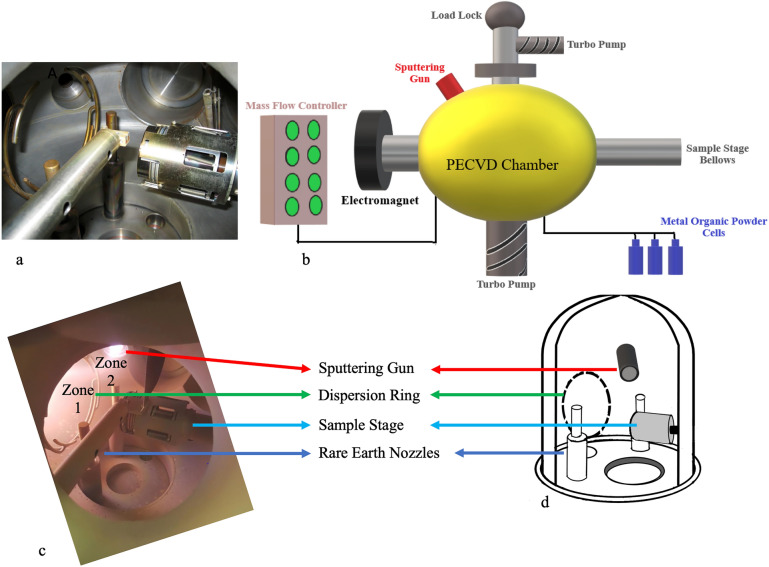

**Supplementary Information:**

The online version contains supplementary material available at 10.1557/s43578-023-01207-2.

## Introduction

Upgrades in semiconductor production technology inevitably are a part of innovation in this information age dominated by telecommunications, green energies, sensors, light-emitting devices [1], lasers [2], and applications in quantum computing allow long coherence lifetime [3, 4]. Silicon (Si), the cornerstone material for microelectronics, is of particular interest for the new generation of photonic devices in one single chip owing to its cost-effectiveness in the use of the existing well-developed technology, complementary metal–oxide–semiconductor (CMOS) technology [5, 6]. Among different approaches used to improve the optical performance of Si-based optoelectronic devices, rare earth (RE) doping is a subject of interest due to RE’s unique characteristic of inter-4f transitions offering a multitude of emissions [7, 8]. RE dopants with very low concentrations (in the order of a few atomic percent) can produce extremely stable, well-defined and sharp emissions with the capability of up-conversion and down-conversion for different light source applications [9, 10]. The optical quality of RE emissions is highly sensitive to the sensitization paths the host matrix provides. Another critical factor determining perspective device performances is the precise control of RE concentration [7]. The RE concentration and the atomic arrangements of the RE ions in the host matrix highly depend on the fabrication/processing technique. We present here the results of RE doping using a custom-made integrated magnetron sputtering and electron cyclotron-plasma enhance chemical vapour deposition (ECR-PECVD) system so-called IMS ECR-PECVD, taking advantage of two common deposition systems and mitigating the weaknesses of many other existing in-situ and ex-situ RE doping methods. The produced thin films showed new and promising properties compared to thin films fabricated using only PECVD. Earlier we published the results of a small subset of samples with fewer details on system configuration [11]. Pauleau et al. [12] reported a hybrid system of microwave PECVD and sputtering for carbon-based structures where 9 at.% of O_2_ contamination the metal is introduced as one of the main components of the film. However, we introduced a hybrid system where sputtering is a replacement for the metal–organic powder techniques to add metals with very low oxygen contamination and controllability from 1 to ~ 17 at.% pushing the solubility of RE doping using common exiting techniques.

Although the 4f-shell transitions of a certain RE ion are determined by the type of RE atom and the structure of the host matrix, the obtained information on the transitions in the host matrix for a certain type of RE ion can predict the transitions of all other types of RE doped in the same host matrix [8]. Here, for terbium (Tb) a proof-of-concept experiment using IMS ECR-PECVD is performed via silicon oxide (SiO_x_) thin films doped with Tb regarding high controllability of the RE dopant concentration and superior quality of the produced samples concerning the light emission properties and material structure. A comprehensive calibration of the system is presented by exploring the influence of the deposition parameters (i.e., sputtering power and argon (Ar) gas pressure) and their influence on the dopant levels. Stoichiometric and non-stoichiometric Tb-SiO_x_ materials were fabricated including terbium-doped silicon dioxide (SiO_2_), silicon-rich silicon oxide (SRSO), and oxygen-rich silicon oxide (ORSO). Using room temperature photoluminescence (PL) spectroscopy, variable angle spectroscopic ellipsometry (VASE), Rutherford backscattering (RBS), transmission electron microscopy (TEM), and X-ray diffraction (XRD), we validated the advantages of this integrated custom-made system that can be commercialized for future semiconductor processing. The correlations between deposition parameters of the sputtering gun embedded in the PECVD chamber and the Tb doping concentration, Tb^3+^ emission, and the nanostructure of the host matrix along with the suggested optimum sputtering power and Ar partial pressure are reported. Furthermore, this technique is not limited to solely dope materials with RE ions; it also can be employed to incorporate boron and other non-RE elements into thin film host matrices to improve electrical, optical, thermal or mechanical properties of the structures showing good potential for solar cell [13] and mechanical applications [14].

## Advantages of an IMS ECR-PECVD system

The idea to combine sputtering and PECVD systems originated from finding a solution to overcome the disadvantages and weaknesses of the already existing deposition methods to dope RE atoms into thin films. Standard techniques to incorporate RE ions include ex-situ and in-situ techniques where the RE ions are incorporated following the deposition and simultaneously with sample growth, respectively. Although the beneficial impact of the ex-situ approach on the control of the dopant level has attracted interest, the damages to the film structure, undesirable defects and intrinsic stresses are the major drawbacks of ex-situ approaches. Ion implantation, one of the commonly used techniques, allows tuning the implant profile accurately along with good reproducibility, however, it suffers from the defects created along the trajectory of ion beam propagation. Regardless of the focusing capability of an ion implanter, the physical limitations of the spatial resolution are unavoidable due to ion straggling and collisions with the atoms of the material [15]. Sputtering and PECVD methods are known as two common in-situ doping methods producing a more uniform distribution of RE dopant and resulting in a higher quality of doped films compared to the ex-situ techniques. Sputtering produces, however, broken bonds and graining effects in the sputtered films compared to the plasma-assisted CVD (PECVD) techniques [16]. PECVD offers the advantages of growing thin films at low deposition temperature, low substrate damage and potentially low damage deposition, controlled and slow deposition rates, and higher quality films, which are all important and independent of specific device structures. On the other hand, the required heating process of Metal–Organic Powders (MOPs) to temperatures as high as 200 °C to vaporize and extract the RE ions into the PECVD chamber makes the control of RE dopant levels more challenging, where undesired cross contamination of the metal–organic powder can be another issue [17, 18].

We designed an upgrade to an existing ECR-PECVD machine to get around the fundamental limit of well-controlled RE concentration in high-quality thin films produced using PECVD depositions. A magnetron sputtering gun is attached to the chamber of the ECR-PECVD system located within the Centre for Emerging Device Technologies (CEDT) at McMaster University. The main advantage of combining a sputtering source and PECVD in one chamber is the accessibility to far greater control of the doping level in a PECVD-grown layer offering higher quality to achieving optically active RE ions.

## IMS ECR-PECVD design

**Figure 1:**
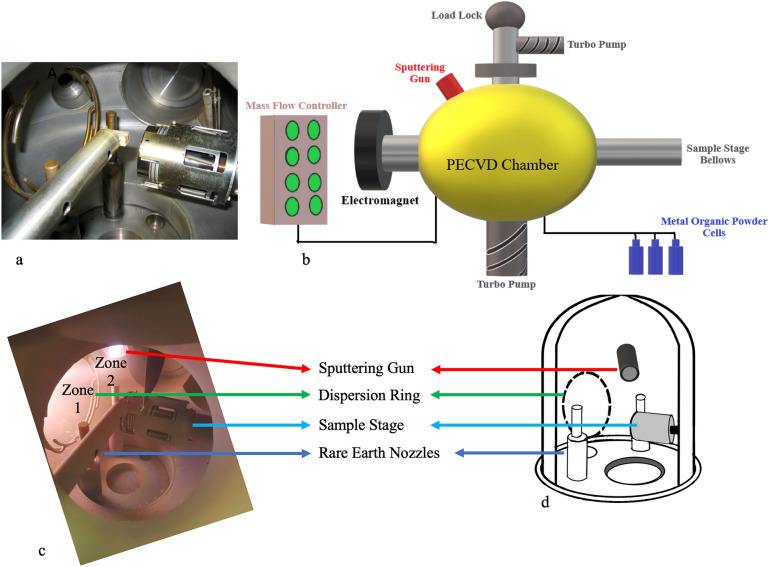
(a) The actual image of the inside of the ECR plasma chamber before the system upgrade with the sputtering source. The dispersion ring, RE nozzles, and sample stage are labelled; (b) The schematic of major external components of the ECR-PECVD system; (c) Active plasma chamber fed with Ar plasma, substrate stage, and sputtering subsystem observed from the ECR front viewport. The typical line-of-sight cosine distribution from the plasma source and the face of the target is identified as Zone 1 and Zone 2, respectively; (d) the schematic of the interior design of the upgraded ECR chamber with the sputtering gun The sputtering gun was positioned passing the dispersion ring in such a way that the sputtering target and the substrate stage face each other at an angle of 30°. The interior components of the system are assigned shared labels.

The ECR-PECVD chamber was upgraded with an axial-mounted high vacuum RF magnetron sputtering gun to incorporate the RE dopants (Fig.1). Technical features of these two subsystems are detailed in the following sections.

### ECR plasma chamber

Prior to attaching the sputtering source, the ECR-PECVD system was installed and designed to allow uniform in-situ doping of RE ions [18]. Figure 1(a) shows the image of the inside of the PECVD plasma chamber during the installation. The major components of the ECR-PECVD system are shown in the schematic diagram of Fig. 1(b): a single toroidal electromagnet generates the required 875 G magnetic field for the electron cyclotron resonance condition using a current of 180 A. The magnet, from one end, is mounted onto the main plasma chamber with a cylindrical configuration, and from the other end is coupled into a water-cooled microwave waveguide absorbing the microwave radiation reflected from the plasma. The magnetron serving as the microwave source is attached to the waveguide with a maximum output power of 1000 W at the commercial band frequency of 2.455 GHz ± 15 MHz. Prior to deposition, the chamber was evacuated to a base vacuum maintained below ~ 0.5 × 10^–7^ using a load-lock chamber and a high-speed Pfeiffer TMH 520 turbo pump with a throughput of 300 L/min. The load-lock chamber decreases the possibility of main chamber contamination upon exposure of the loading chamber to the atmosphere [19]. Four lines of volatile metalorganic precursors deliver several RE dopants and are heated in the range of 170 to 250 °C in a canister enveloped in a customized jacket to sublime the precursor. The metal vapours are carried from the canister to the plasma chamber via nozzles using Ar as the carrier gas. The metalorganic compound RE(tmhd)_3_ or tris (2,2,6,6-teramethyl-3,5-heptanedionato)-RE(III) was used as the RE source that was fed into the chamber alongside the precursors of 30% SiH_4_ in Ar and 10% O_2_ in Ar as the Si and oxygen (O) sources, respectively. All gases are delivered through 1/4″ type 304 stainless steel tubes using mass flow controllers in the unit of “standard cubic centimetres per minute” (SCCM), where standard refers to the standard conditions for temperature and pressure. The ECR chamber was designed to feed diluted O_2_ gas into the plasma region and supply diluted SiH_4_ gas downstream from the discharge zone through a dispersion ring, as labelled in Fig. 1(c) and (d), placed out of the plasma region at zero angles to the substrate. The sample stage is heated to a temperature of 120 °C at the substrate resulting from a stage heater set to 350 °C. The capability of continuous stage rotation at approximately 25 rotations per minute provides high uniformity and homogeneity of the RE ions and other constituent elements such as Si and O through the depth of the deposited layer.

### Sputtering subsystem

A picture of the final configuration for the sputtering system attached to the ECR chamber is shown in Fig. 1(c) and the practical design considerations are labelled. The typical line-of-sight cosine distribution from the plasma source and the face of the target is identified as Zone 1 and Zone 2, respectively. The target-to-substrate distance is one of the factors determining the RE dopant levels, and on the other hand, increased distance controls the target poisoning [20]. The magnetron sputtering gun with its shutter is calibrated to be spaced 16.5 cm from the sample stage at an angle of 30°. The arrangement enables more uniform distribution of incident flux on the sample stage placed at the focus of the angled sputtering gun. The single-element target with 2″ diameter X 0.125″ thick disks and a purity of 99.9% of the chosen material, in this study Tb, was employed. To avoid target melting, the sputtering guns are actively water-cooled which is extra protected by bonding copper to the back of the target (Supplementary Material Fig. S1.). The inert gas (Ar) is directly fed to the working atmosphere close to the sputtering head by a mass flow controller different from the one used for the generation of ECR plasma. The Ar glow discharge generated between the sputtering target (99.9% Tb) and the sample stage is shown as Zone 2 in Fig. 1(c). Radiofrequency (RF) is used to ignite the plasma in the vicinity of the sputtering gun and knock the RE atoms from the sputtering target. The 13.56 MHz RF power generator by far is the most used frequency in industrial sputtering applications [16]. The range of the sputtering power is set from 10 W to a maximum of 60 W to avoid overheating of the target. In the absence of copper plates, the operation has to be performed at low sputtering power below 20 W due to the slow heat transfer.

## Results

In exploring the functionalities of the sputtering source embedded in the ECR-PECVD chamber, the system calibration was primarily performed by investigating the influence of the sputtering power and Ar gas flow on the Tb doping level. Due to the large number of samples and the volume of collected data from RBS, we employed machine learning techniques to capture the relationship between the Tb dopant concentration for three categories of film compositions including oxygen-rich, silicon-rich and stoichiometric SiO_x_. In addition to the Tb concentration, we investigated the optical properties of all three stoichiometries of Tb-SiO_x_ including refractive indices, optical band gap, and PL. The correlation of the sputtering power and the dopant concentration was found to be different in the three types of SiO_x_ compositions and depended on the oxygen content in the thin film. The results of Tb-SiO_x_ samples can be compared with previously reported samples fabricated using the same ECR-PECVD machine with in-situ doping of RE elements via the heating of volatile metal–organic compounds, RE(tmhd)3 or tris (2,2,6,6-teramethyl-3,5-heptanedionato)-RE(III) [21, 22].

### Role of argon and power on terbium dopant level

As opposed to the other measurements for which we employed a single side polished Si substrate, the use of graphite substrate for the RBS compositional analysis avoids the interference of the Si signal of the substrate with elements lighter than Si, therefore, it delivers an accurate calculation of the dopant level in the order of 0.1 at.%. [23]. A typical experimental RBS spectrum and the fit generated by the SIMNRA program with the table of the elemental concentration of all samples are provided in Supplementary Materials, Fig. S2.

**Figure 2:**
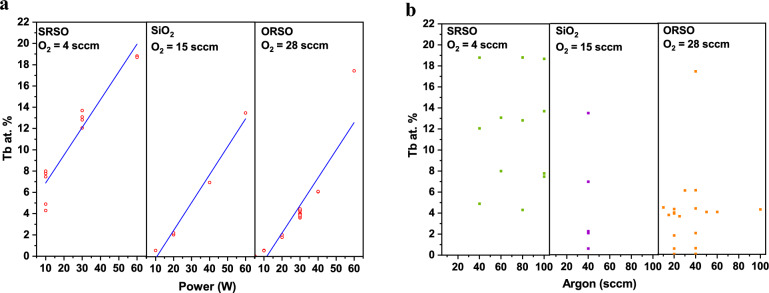
The estimated correlation between the Tb concentrations (at.%) and the increase of the sputtering power and Ar gas flow is modelled. To isolate the cases for different oxygen contents, the three different parts are divided by the oxygen values 4, 15 and 28 sccm representing SRSO, SiO_2_, and ORSO matrices, respectively; (a) The Tb concentrations (at.%) increase with the increase of the sputtering power. The scope of the model is in the range of the power values as the independent variable from 10 to 60 W; (b) Ar does not play a considerable role in the dopant concentration. The only minor influence of Ar flow is observed at low power regions in that with the use of higher flow rates of Ar, higher Tb content is incorporated in the film.

**Figure 3:**
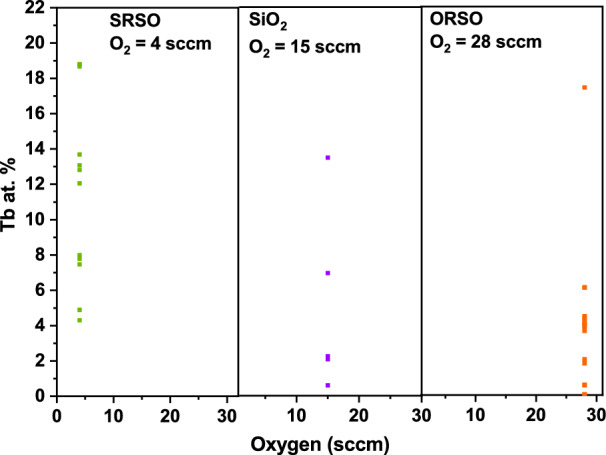
The variation of the Tb concentration for different film compositions grown using three different O_2_ gas flow rates. The dots represent the Tb content of SiO_x_ matrices fabricated using a constant value of SiH_4_ gas flow. The incorporation of more Tb in the SRSO (silicon-rich SiO_x_) thin films is shown. The lowest (highest) dots on the vertical axes in each range of the graph correspond to the lowest (highest) sputtering power, 10 W (60 W).

With the use of machine learning, multiple linear regression models were developed for different regimes based on the O_2_ value that represents the relationship between the variation of the sputtering powers or Ar flow and the Tb concentrations (at.%) as shown in Fig. 2. To isolate the cases for different O contents, the three different parts are divided by the O_2_ values 4, 15 and 28 sccm, representing SRSO, SiO_2_, and ORSO matrices, respectively. The parameters of the linear regression models are provided in the Supplementary Material for those three regimes. The Pearson correlation coefficients are 0.9673, 0.9927, and 0.9163 for O_2_ gas values of 4, 15, and 28 sccm, respectively verifying the validity of the learned models. The Tb concentration linearly increases with the increase of the sputtering power for all silicon-rich, oxygen-rich, and stoichiometric SiO_x_ matrices. On the other hand, the variation of Ar gas flow only slightly changes the dopant concentration. The only minor influence of Ar flow is observed at low power regions in that with the use of a higher flow rate of Ar, higher Tb content is incorporated in the film. The comparison of the starting points of the fitted lines of three different compositions of SiO_x_ in Fig. 2(a), shows that identical values of sputtering power and Ar gas flow rate produce more Tb-rich structures in SRSO thin films compared to stoichiometric SiO_2_ and ORSO thin films, i. e., when, with the increase of O_2_ gas flow rate, the SiO_x_ host matrix moves towards the O-rich structure, a lower amount of Tb is incorporated in the film. This is shown more clearly in Fig. 3. presenting the variation of the Tb dopant concentration for different film compositions grown using three different O_2_ gas flow rates. This is in contrast to samples fabricated using metal organic powders and ECR-PECVD that incorporated more Er or Tb ions with the increase of O [24].

**Figure 4:**
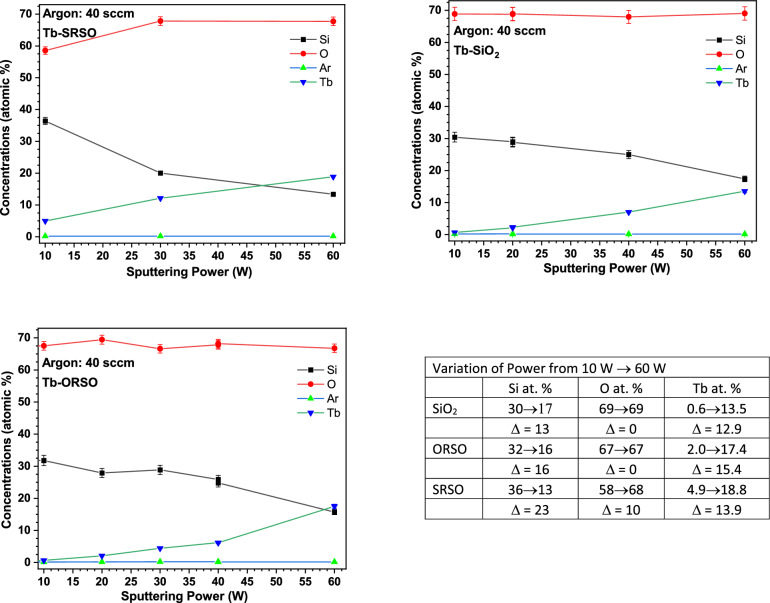
Elemental concentration of all three types of SiO_x_ thin films extracted from the RBS analysis. In all compositions, the increase of Tb is compensated by a loss of Si. O content only increases in the SRSO (silicon-rich SiO_x_) thin films, which results in a greater loss of Si compared to that in ORSO (oxygen-rich SiO_x_) and SiO_2_ matrices.

Concerning the variations of other elements, i.e., Si and O, Fig. 4 shows the variation of the concentration of all constituent elements of three types of Tb-doped SiO_x_ compositions fabricated with an Ar flow rate of 40 sccm as a function of the sputtering power, with the corresponding experimental uncertainties extracted from RBS analysis. The changes of the atomic percent of each element with the variation of the power and Ar gas flow are tabulated in Fig. 4. In all SiO_x_ compositions, the increase of Tb with the increase of power is compensated by a loss of Si of about ~ 13 to 23 at.% in the layer. The O content remains nearly constant except for SRSO where there is a slight increase of O (~ 9 at.%), which results in a greater loss of Si and does not necessarily lead to the further incorporation of Tb. Both in the low- and high-power regions, the incorporation of Tb into the SiO_2_ matrix is smaller than in ORSO or SRSO.

### Optical constants

**Figure 5:**
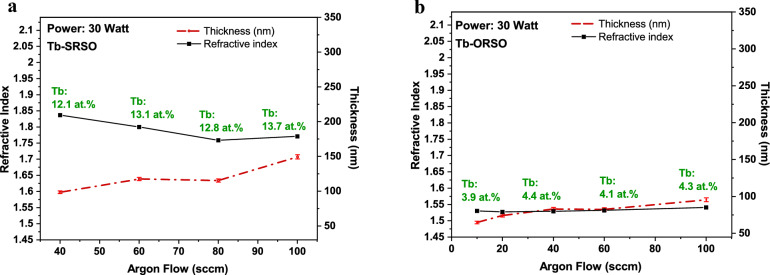
(a) and (b) Dependence of the refractive index (n) and thickness on Ar flow rates for ORSO and SRSO samples grown at a fixed sputtering power of 30 W.

**Figure 6:**
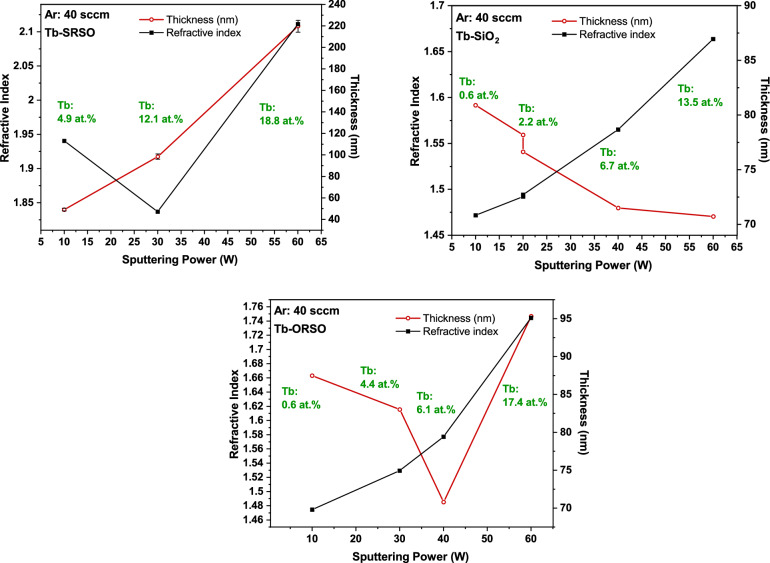
The influence of the sputtering power on the refractive index (n) and thickness of ORSO, SRSO, and SiO_2_ samples grown using a constant Ar gas flow rate of 40 sccm. Independent of the composition of SiO_x_ the refractive index (black solids lines) increases with the increase of power. In contrast, the thickness (red dot lines) increases in silicon-rich samples while it decreases in oxygen-rich and stoichiometric silicon oxide (ORSO and SiO_2_).

In the absence of Tb metal atoms in the structure of transparent SiO_x_, a Cauchy model is commonly employed for the simulation of VASE data in the visible region. The presence of Tb introduces absorptions in the visible and NIR regions requiring the use of alternative models. Here, a B-Spline model was used for the fitting of the VASE data to describe the dispersion relation delivering the refractive index (n) and film thickness as a function of wavelength (λ). To better understand the variation of the thickness and refractive index as a function of sputtering power and Ar gas flow for different compositions of SiO_x_, Figs. 5 and 6 are plotted on identical scales representing a range of 1.45 to 2.1 for the refractive index and film thicknesses of 50 to 340 nm. To distinguish the changes induced by Tb doping from the changes resulting from the host SiO_x_ matrix, the values of the undoped SiO_x_ matrices are listed in Table 1 along with the observed behaviour of the refractive index and thickness of the Tb-doped matrix as a function of the sputtering power and Ar flow rate. ORSO and SiO_2_ structures are low refractive index materials (~ 1.5) while the refractive index of SRSO is greater due to the presence of excess Si in the matrix. Table 1 shows that the use of a higher O_2_ gas flow rate to produce the ORSO matrix makes the deposited layer significantly thinner (less than half thick of stoichiometric SiO_2_), while the excess Si in the structure of SRSO resulting from the use of lower O_2_ gas flow rate during the deposition does not change the thickness compared to that of SiO_2_. The increase of the O_2_ gas flow rate during the deposition brings in multiple factors such as different growth rates and different reactions at the surface changing the film composition, density, and hydrogen content. The increase of O_2_ decreases the ratio of SiH_4_/O_2_ and SiH_4_, the only source of hydrogen in the layer. Lower hydrogen content in the film structure leads to a denser layer and less porosity.

**TABLE 1:** The variation of the refractive index and thickness of ORSO and SRSO samples grown at a fixed sputtering power of 30 W.

Refractive Index	Undoped	Tb-doped with the increase of power	Tb-doped with the increase of Argon
SiO_2_	1.47	0.2 increase	–
ORSO	1.45	0.3 increase	0.03 decrease
SRSO	1.81	0.06 increase	0.07 decrease
Thickness (nm)	Undoped	Increase of power	Increase of Argon
SiO_2_	68	~ constant (10 nm decrease)	–
ORSO	73	~ constant (7 nm decrease)	30 nm increase
SRSO	41	170 nm increase	50 nm increase

#### Refractive Index

*Argon role* Figure 5 shows the observed trend of the changes in the refractive index and film thickness as a function of the increase of Ar flow rate from 10 to 100 sccm. The refractive index is virtually constant with the increase of Ar flow for all three types of host matrices, which could be expected from the close values of Tb concentrations in these three matrices fabricated with a fixed sputtering power. The Lorentz-Lorenz equation explains how the refractive index varies with both the changes in the atomic configuration and the density of the films, being correlated parameters [25]. Likely the decrease in density of the films with the use of a higher Ar flow rate is responsible for the slight decrease of the refractive index since the Tb content (and therefore the material composition) does not change significantly with the variation of the sputtering Ar gas flow.

*Power role* Figure 6 shows that the refractive index increases with the increase of power for all Tb-doped ORSO, SRSO, and SiO_2_ compositions, which can be explained in terms of the increase of the Tb content in the structure. In the Supplementary Materials, Fig. S3 presents more plots showing an increasing trend with increasing sputtering power at fixed values of Ar gas flow. There are two competing factors in the variation of the refractive index; the addition of heavy elements such as Tb into the structure increases the bonding strength of the SiO_x_ (glass) structure and therefore increases the refractive index; while the second factor, Si loss, contributes to a lower refractive index. The Si loss was observed in RBS compositional analysis showing Tb atoms mainly substituting for Si atoms rather than O atoms). The rate of the increase of the refractive index of SRSO with increasing power (higher Tb content) is smaller than that of ORSO and SiO_2_.

#### Thickness

In contrast to the similar trends observed for the changes of refractive index for all host matrices, the thickness of Tb-SRSO samples decreases with the increasing sputtering power, compared to ORSO and SiO_2_ compositions where the thickness is nearly constant (decrease of only a few nanometres). The significant thickening of SRSO from 50 to 220 nm with the increase of the sputtering power from 10 to 60 W occurs with a significant Si loss and increase of O content and significant hydrogen increase as observed with elastic recoil detection (EDR) measurements that are not discussed in this work (Fig. 4). With the increase of power, the SRSO matrix moves from Si-rich to O-rich structure which is nearly doubly as thick. The values of undoped reference samples in Table 1 show that the undoped ORSO and SiO_2_ structures with a thickness of about 70 nm are thicker than undoped SRSO structures with a thickness of about 40 nm. Variation of the Ar flow rates leads to increases in the layer thickness for all three types of samples.

### Photoluminescence (PL)

To activate the luminescent emission of the Tb ions, all three sets of Tb-doped samples were subjected to post-deposition thermal annealing in an ambient of nitrogen and forming gas (%95 N_2_ + %5 H_2_) for 1 h using a quartz tube furnace at 1200 °C. Optical activation of the Tb ions was studied primarily through room temperature ultraviolet-excited PL detected in the visible range, which provided a means of measuring the sensitization of Tb ions in the SiO_x_ host matrix over a broad range of sputtering conditions i.e., sputtering powers and Ar flow rates for all three subsets of Tb-SiO_x_ thin films.

Typically, the samples show four PL peaks located at 490, 545, 590, and 624 nm associated with the ^5^D_4_ → ^7^F_j_ transitions of trivalent Tb (Tb^3+^) where *j* = 6, 5, 4, 3, respectively [8]. All these peaks have previously been observed in Tb-SiO_x_ matrices fabricated by ECR PECVD and the use of metalorganic powders [21] and sputtering methods [26]. To compare the PL intensity of different samples we considered the strongest emission peak centred at 545 nm (^5^D_4_ → ^7^F_5_) which is the technologically most important transition. The influence of power (and to some extent Ar flow) on O-rich samples was found to be different from that on silicon-rich samples as will be discussed in the following subsections.

#### Role of power on the PL of Tb:ORSO

**Figure 7:**
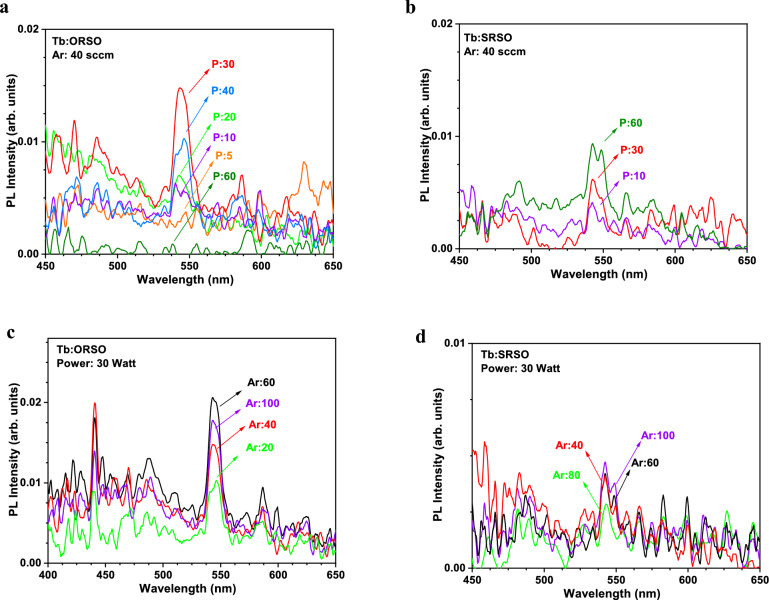
The influence of power on the PL emission of Tb^3+^ ions in ORSO (a) and SRSO (b) samples grown by sputtering at an Ar flow rate of 40 sccm; (c) and (d) The role of Ar gas flow in the PL emission of Tb^3+^ ions in ORSO (c) and SRSO (d) samples fabricated using a fixed power of 30 W.

Figure 7(a) shows PL spectra for O-rich samples fabricated with identical deposition parameters except for different sputtering powers, ranging from 5 to 60 W. To verify the observed trend, in the Supplementary Material, Fig. S4, a graph similar to Fig. 7(a) shows the role of power on the PL of ORSO samples using a fixed Ar gas flow rate of 20 sccm. The optimum PL emission from the activation of Tb ions was achieved at *P* = 30 W. Although a further increase of the power introduced more Tb ions in the matrix, it degrades the PL intensity. The increase of the PL emission up to *P* = 30 W can be explained in terms of the increase in dopant density and consequently the increase in the possibility of RE sensitization through O-related defects [22]. The degradation of the PL intensity at sputtering powers exceeding 30 W is associated with a strong tendency of RE ions to cluster together as observed in the TEM images of the highly doped ORSO sample (Tb-ORSO-Ar40P60). RE clustering increases the probability of non-radiative de-excitation pathways due to undesired ion-ion interactions [7]. This cluster formation in the Tb-rich structure and its detrimental effect on the optical activation of Tb^3+^ ions has been commonly observed in other RE-doped silicon-based structures [26]. The broad peaks at the shorter wavelengths (higher energies) are related to the structure of the host matrix coming from defect-related and/or band-tail states within the confined regions of the oxygen-rich or silicon-rich regions [10, 21, 22].

#### Role of power on the PL of Tb:SRSO

Figure 7(b) demonstrates that the increase of the power leads to a continuous increase in the PL intensity of SRSO samples, in contrast to the ORSO matrix where there was a threshold for the sputtering power to enhance the PL emission. In the Supplementary Material, a similar trend of the continuous increase of the PL emission with the increase of power is observed for SRSO samples fabricated by Ar = 80 sccm (Fig. S5). It should be noted that the different luminescent behaviour of Tb:SRSO and Tb:ORSO with increasing sputtering power is despite the incorporation of the virtually identical Tb concentrations and identical sputtering parameters. For example, ORSO and SRSO samples fabricated with the highest power (Tb-SRSO-Ar40P60 and Tb-ORSO-Ar40P60) contain 18.8 at.% and 17.4 at.%, respectively, however, in the ORSO sample (Tb-ORSO-Ar40P60), all 4f-transitions of Tb ions are quenched. The coordination of the Tb ions in the SRSO structure is different from the ORSO matrix in such a way that the lack of O in the SRSO matrix prevents the ion-ion interactions from quenching the PL emission. From the above results, we suggest a power range of 30 to 40 W to efficiently activate the transitions of trivalent Tb ions in ORSO matrices and further increase the sputtering power (incorporating more Tb in the film) caused the PL decay. In contrast, the increase of the power increases the PL intensity of SRSO samples, and no saturation of the PL increase was observed, so the maximum power of 60 W to achieve heavily doped SRSO samples with the highest luminescent is suggested.

#### Role of Ar on the PL of Tb:ORSO

Figure 7(c) shows the dependence of the PL on the Ar flow for ORSO samples where a fixed sputtering power of 30 W was used. First, by increasing the Ar gas flow from 20 to 30 sccm, the intensity of the PL peak increases for sample Tb-ORSO-Ar30P30, then gradually decreases with a further increase of Ar flow, so that the most intense Tb^3+^ luminescence from 5d to 4f states occurred with the use of an Ar flow of 60 sccm. The maximum PL intensity did not vary significantly for any Ar gas flow rate higher than Ar = 60 sccm. The insignificant influence of Ar variations on the PL intensity could have been anticipated from the virtually identical values of Tb concentrations for these ORSO samples fabricated using a fixed value of sputtering power, e.g., the use of 30 W power for Tb-ORSO-Ar20P30, Tb-ORSO-Ar30P30, Tb-ORSO-Ar100P30, Tb-ORSO-Ar60P30, Tb-ORSO-Ar40P30 resulted in 3.9, 4.3, 4.2, 4.1, 4.3 at.% of Tb. A similar trend of a negligible role of Ar flow was observed for samples annealed at 1200 °C using forming gas of N_2_+H_2_ (instead of pure N_2_), which suggests the optimum Ar gas flow ranging between 30 to 60 sccm for the deposition of Tb-doped ORSO.

#### Role of argon on the PL of Tb:SRSO

Figure 7(d) shows that Ar gas flow does not play any considerable role in the PL emission of the SRSO samples similar to its insignificant role on the Tb dopant level (section 5.1). However, when the power is as high as 60 W (Supplementary Material, Fig.S6), the use of Ar overpressure to the maximum capacity of the mass flow controller (such as Ar:100 sccm) decreases the PL intensity. Therefore, we suggest using an Ar flow to a maximum 60 sccm. Similar behaviour was observed for ORSO, where up to Ar = 60 sccm there was no effect on the PL emission. The comparison of power increase for samples fabricated using medium and high range of Ar flow suggests using an Ar flow between 40 to 60 sccm for the SRSO samples.

#### Comparison of the influence of power for low and high Ar flow rates

The influence of the sputtering power on the Tb-doped samples fabricated with different Ar gas flows suggests that in the lower power regions (any power below 20 W) emission from trivalent Tb is not optically activated when the Ar flow is low. With the increase of Ar flow to 40 sccm (Fig. 7(a)), the influence of low power is less significant, and more Tb ions are optically activated despite similar Tb content. This indicates that the use of a higher Ar flow rate allows using a lower sputtering power to achieve certain Tb content. We suggest Ar gas flow rates in the range of 30 to 60 sccm for all types of RE-doped SiO_x_ and all ranges of sputtering power.

#### Role of Ar flow in the ECR plasma

**Figure 8:**
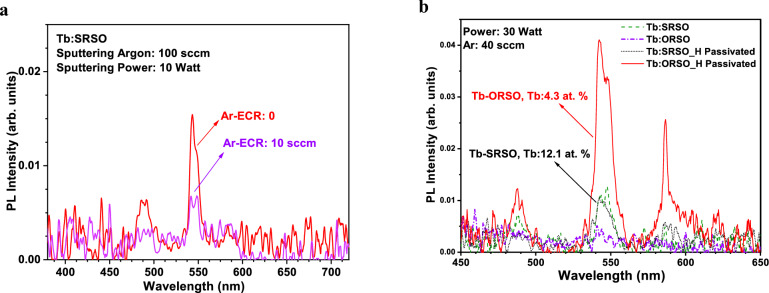
(a) The influence of Ar gas flow in the ECR-PECVD plasma on the PL emission of Tb; (b) comparison of the PL spectra of passivated and non-passivated Tb:SRSO and Tb:ORSO fabricated using identical deposition parameters.

Figure 8(a) shows Tb-SRSO samples fabricated using identical parameters except for the labelled sample where an additional 10 sccm of Ar gas flow was used for the generation of the ECR-PECVD plasma (Zone 1, Fig. 1(c)). The increase of Ar in the plasma decreases the intensity of the PL emission. This is likely due to the slight decrease of Tb concentration with the use of Ar gas in the PECVD region which decreases the density of Tb ions to be sensitized through defects or nanocrystals formed in the Si-rich regions.

#### Comparison of Tb:SRSO and Tb:ORSO for the suggested optimum parameters

Due to the large volume of data, the comprehensive analysis of the PLof hydrogen passivated is not discussed and only a representative of Tb:ORSO and Tb:SRSO samples fabricated with the suggested optimum sputtering parameters and annealed using N_2_H_2_ is shown in Fig. 8(b). The use of forming gas enhances the distinct emission from 5D to 4F transitions and makes the signature double-peak emission of Tb^3+^ emissions more distinguishable in both SRSO and ORSO. The point to consider is that using identical sputtering parameters produces highly doped SRSO structures (~Tb: 12.1 at.%) while the dopant concentration in ORSO structures is much lower (~Tb: 4.3 at.%) The intensity of PL emission is, however, nearly equal for both SRSO and ORSO samples. The hydrogen passivation during annealing at 1200 °C significantly improves the PL emission of Tb-doped ORSO. The discussion of the underlying mechanism is out of the scope of this work. For optical activation of Tb^3+^, we suggest the use of ORSO structures with post-deposition thermal annealing in forming gas.

### Nanostructures

**Figure 9:**
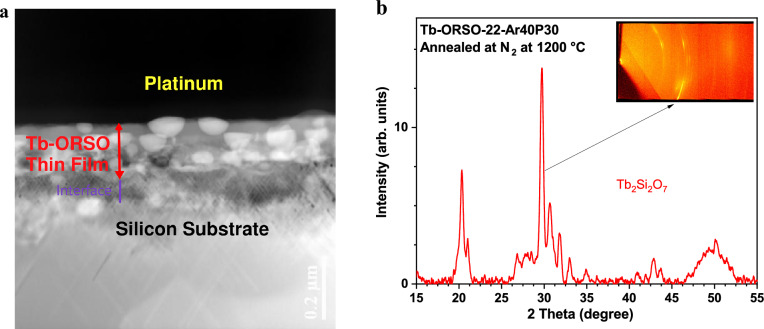
Tb-ORSO-22-Ar40P30 thin film annealed at 1200 ℃ for 1 h in N_2_ atmosphere; (a) High-resolution TEM cross-sectional image of FIB-prepared lamella showing nanocrystals embedded in the amorphous surrounding SiO_x_ matrix; (b) 2D diffraction pattern with a phase identified as Tb_2_Si_2_O_7_.

Figure 9 shows a cross-sectional HRTEM image, and 2D XRD patterns taken from Tb-ORSO-Ar40P30 containing a Tb concentration of 4.4 at.% and annealed in N_2_ enviroement at 1200 °C for 1 h. In contrast to ORSO samples, none of the Tb-SRSO and Tb-SiO_2_ samples produce diffraction patterns and any nanocrystal phases which confirms the amorphous structures of Tb-SRSO and Tb-SiO_2_ samples lacking long-range crystallographic order. In Fig. 9(a) the red arrow shows the deposited layer of Tb-ORSO (Tb: 4.4 at.%) with a thickness of about 120 nm grown on top of the crystalline Si substrate. The Pt layer is the protective layer of the FIB-prepared sample. The bright-field HRTEM image clearly shows the significant restructuring and the formation of nanocrystals of the Tb-disilicate structure at 1200 ℃. Figure 9(b) is the image of the flattened 2D pattern generated by the general area detector diffraction system (GADDS). The grain XRD analysis supports the findings of HRTEM in that the phase analysis reveals a polycrystalline structure with the presence of large crystal grains of Tb_2_Si_2_O_7_ embedded in the amorphous SiO_x_. The highest diffraction peak in the simulated 2D pattern corresponds to the (− 4, 0, 2) plane which is presented by discrete lines in the diffraction map as shown in the inset of Fig. 9(b). To the best of the author’s knowledge, this work is the first report on the Tb-disilicate formation for samples fabricated by an IMS ECR-PECVD system.

## Conclusions

We established a highly effective method to control rare earth (RE) doping of thin films and reported the first comprehensive study on the influence of sputtering conditions on the structure and properties of Tb-doped glasses (Tb-SiO_x_). The customized IMS ECR-PECVD machine combines the advantages of the PECVD method for the fabrication of host matrices with controlling the RE doping by an integrated sputtering gun attached to the PECVD plasma chamber. The study of Tb-doped SiO_x_ matrices covering the entire range of stoichiometric and off-stoichiometric compositions, including O-rich and Si-rich SiO_x_ shows that for all Si-rich, O-rich and stoichiometric SiO_2_ matrices with the increase of the sputtering power, rare earth dopant level increases with virtually similar increase rate calculated by the machine learning modelling for a large number of samples. On the other hand, Ar gas flow was found to only be slightly effective in the increase of the dopant concentrations at low power. Identical values of sputtering power and Ar gas flow rate produce more Tb-rich samples in SRSO thin films compared to stoichiometric SiO_2_ and ORSO samples. The refractive index regardless of the type of the matrix increases with the increase of power for all ORSO, SRSO, and SiO_2_ compositions and is virtually constant with the increase of Ar flow for all three types of host matrices. The near independency on Ar flow was expected from the close values of Tb dopant concentrations in these three matrices fabricated with a fixed sputtering power and varying Ar flow. The insignificant influence of Ar and the dominant role of the power was found to be similar for O-rich SiO_x_ and stoichiometric SiO_2_ in contrast to Si-rich samples where their thickness greatly increases with the increasing power.

Based on the optical activation of Tb^3+^ emissions, we suggest Ar gas flow rates in the range of 30 to 60 sccm (corresponding to partial pressures between 3.3 to 4.2 mTorr) for all types of RE-doped SiO_x_ and all ranges of sputtering power. The fabrication of SRSO or ORSO depends on the purpose of the study as each matrix offers advantages, however, when the PL emission of RE is the main concern an O-rich matrix is suggested. RE doping in the glass structure as high as ~ 20 at.% was achieved (compared to the use of heating cells for the metalorganic powders limiting the RE dopant to less than 10 at.%). The system calibration alongside optimizing the system-specific process parameters establishes foundational knowledge that promotes future work on doped- Si-containing thin films using the proposed novel integrated sputtering and PECVD (IMS ECR-PECVD) system. If multiple dopants are of interest, the co-doping of RE using both one-element sputtering target and metalorganic powders in the PECVD, where in principle, the magnetron sputtering and each metalorganic serves as one source of the RE material, is offered for future nanophotonic materials, light emitting devices (LEDs), solar cells, and quantum memories.

## Methodology

### Deposition design

To establish the process parameters of the IMS ECR-PECVD system, Tb-doped silicon-rich and oxygen-rich silicon oxide thin films, so-called Tb-SRSO and Tb-ORSO, respectively, as well as Tb-doped stoichiometric silicon dioxide (Tb-SiO_2_) were fabricated with a wide range of sputtering powers and Ar partial pressures. The thin films were fabricated on p-type crystalline Si < 100 > using a mixture of 30% SiH_4_ in Ar and 10% O_2_ in Ar with a constant gas flow rate of SiH_4_ at 2 ± 5% sccm corresponding to a partial pressure of 2.5 mTorr. The use of 4, 15, and 25 sccm of 10%O_2_/Ar resulted in the growth of SRSO, SiO_2_, and ORSO thin films, respectively. The values of the partial pressure of the O_2_ gas source and all other deposition parameters for all samples are listed in Table S1, Supplementary Material. Thin films were deposited in 1 h at a nominal deposition (stage) temperature of 350 °C corresponding to the substrate temperature of approximately 120 °C. The depositions were performed using a fixed microwave power of 500 W to generate the plasma in the PECVD chamber. RF sputtering power varying from 10 to 60 W was employed to sputter Tb from the target towards the growing layer on the sample stage, with Ar partial pressures ranging from 2.2 to 8.9 mTorr, corresponding to gas flow rates of 20 to 100 sccm. To purify the sputtering target, it was sputtered for 2 min using a maximum sputtering power of 60 W without opening the shutter of the sample stage. In addition to a few undoped SiO_x_ samples as references, 37 SiOx thin films of different stoichiometries were fabricated with an extensive range of dopant levels ranging from 0.5 to 18 at.%.

### Samples labelling

To ensure reproducibility of the fabrication recipe on another setup and transferability of the method in general, the sputtering power densities and gas partial pressures have to be reported, rather than sputtering powers and gas flow rates. In this work, we used a harmonization, as presented in Table 2, to facilitate a smoother reading/modelling of a large data set and to accurately understand the correlations, where “power” instead of “sputtering power density” and “Ar gas flow” instead of “sputtering argon gas partial pressure” is used. Accordingly, sample labelling in the form of, “Tb-SRSO or ORSO or SiO_2_-(values of Ar)Ar-(values of power)-P” has been employed. The full list of all 37 samples, their deposition parameters, and concentrations of Tb dopant are tabulated in Table 1 of the Supplementary Material.

**TABLE 2:** The range of the sputtering power and corresponding power density on the Tb sputtering target and the range of sputtering Ar gas flow with the corresponding partial pressures.

Power (W)/power density (W/cm^2^)	10/0.5	20/1	30/1.5	40/2	60/3	–
Ar Gas Flow (sccm)/Ar Partial Pressure	20/2.2	30/3.3	40/3.2	60/4.2	80/7.7	100/8.9

### Characterization techniques

Rutherford backscattering spectrometry (RBS) was employed to determine the thin film composition, using a facility located at the Tandetron Laboratory at Western University, London, Ontario. Thin films were bombarded by 1.8 MeV 4He^+^ ions with detectors placed at a scattering angle of 170°. The room-temperature PL spectra of the samples were measured using a continuous-wave 325 nm He-Cd laser (*E*_exc_ = 3.82 eV) with an optical power of 5 mW exciting an area of 2.3 mm^2^. Using objective lenses, the PL spectra were collected and focused on a fibre optic spectrometer 2000 [27]. The recorded room-temperature PL data as a function of wavelength was first corrected with the response of the system, then a spectrum of the empty stage with no sample on it was subtracted to apply additional background subtractions using custom code developed with Matlab [28]. Variable angle spectroscopic ellipsometry (VASE) measurements were obtained using a Woollam M-2000 VASE system through the reflection spectra at multiple angles of incidence (55°, 60°, 65°, 70°, and 75°) in the range of UV–VIS–NIR (300–1600 nm). The Ψ and Δ values delivered directly from the VASE data were analyzed by the CompleteEASE software (J.A. Woollam Inc) to determine the refractive indices (n) and film thicknesses [29]. To identify the crystal phase of the samples powder X-ray diffraction (XRD) patterns of the thin films were evaluated with a Cu K_α_ source in a Bruker D8 Davinci X-ray diffractometer. The nanostructure of Tb-SiO_x_ thin films was investigated at the Canadian Centre for Electron Microscopy (CCEM) at McMaster University, using transmission electron microscopy (TEM) using an FEI Titan operated between 80 to 300 keV with a theoretical point-to-point spatial resolution of 0.20 nm at 300 keV. The TEM samples were prepared by focused ion beam (FIB) where the area of interest (lamella) was protected by the deposition of a platinum (Pt) layer.

### Supplementary Information

Below is the link to the electronic supplementary material.

Supplementary file1 (DOCX 695 KB).

## Data Availability

N/A.
