# Supplementary material for: A comprehensive calibration of integrated magnetron sputtering and plasma enhanced chemical vapor deposition for rare-earth doped thin films
Source: J Mater Res. 2023 Nov 7;39(1):150–64. doi: 10.1557/s43578-023-01207-2 (PMC10784389; doi:10.1557/s43578-023-01207-2)
Supplement: Supplementary file 1 — Supplementary file1 (DOCX 695 KB). [file 43578_2023_1207_MOESM1_ESM.docx]

**Supplementary Material**


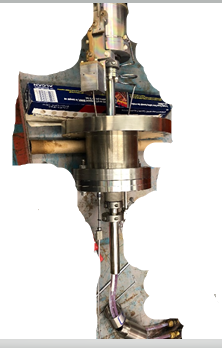


Fig. S1. The image of the whole set up of the sputtering gun and its schematic. To avoid target melting, the sputtering guns are actively water-cooled.

Table S1. The values of all other deposition parameters for all samples including the partial pressure of the O_2_, SiH_4_, and Ar sputtering gas sources and the dopant level in the deposited film. To produce the three stoichiometric of SiO_x_, SiH_4_ was kept constant at 2 sccm and O_2_ gas source was set at 4, 15, and 25 sccm to produce SRSO, SiO_2_, and ORSO matrices, respectively.

| **Sample** | **Tb**  **at. %** | **30%SiH_4_**  **[sccm]/**  **mTorr** | **10%O_2_**  **[sccm]/**  **mTorr** | **Sputtering**  **Ar**  **[sccm]/**  **mTorr** | **Sputtering**  **Power**  **[W]** | **Power Density [W/cm^2^]** |
| --- | --- | --- | --- | --- | --- | --- |
| SiO_2_ | **-** | 2/0.25 | 15/1.76 | 0 | 0 | 0.0 |
| SRSO_1 | **-** | 2/0.25 | 4/0.5 | 0 | 0 | 0.0 |
| SRSO_2 | **-** | 2/0.23 | 4/0.46 | 0 | 0 | 0.0 |
| SRSO_3 | **-** | 2/0.26 | 5/0.66 | 0 | 0 | 0.0 |
| ORSO_1 | **-** | 2/0.23 | 28/3.05 | 0 | 0 | 0.0 |
| Tb-SRSO_1_Ar80P10 | **4.30** | 2/0.25 | 4/0.5 | 80/7.7 | 10 | 0.5 |
| Tb-SRSO_2_Ar80P30 | **12.82** | 2/0.3 | 4/0.57 | 80/7.9 | 30 | 1.5 |
| Tb-SRSO_3_Ar80P60 | **18.81** | 2/0.29 | 4/0.55 | 807.9 | 60 | 3.0 |
| Tb-SRSO_4_Ar100P10 | **7.78** | 2/0.22 | 4/0.44 | 100/9.0 | 10 | 0.5 |
| Tb-SRSO_5_Ar100P30 | **13.69** | 2/0.24 | 4/0.45 | 100/9.4 | 30 | 1.5 |
| Tb-SRSO_6_Ar100P60 | **18.69** | 2/0.25 | 4/0.5 | 100 | 60 | 3.0 |
| Tb-SRSO_7_Ar100P_ArPlasma10 | **7.48** | 2/0.28 | 4/0.52 | 100/10.3 | 10 | 0.5 |
| Tb-SRSO_8_Ar60P10 | **7.99** | 2/0.28 | 4/0.52 | 60/6.12 | 10 | 0.5 |
| Tb-SRSO_9_Ar60P30 | **13.09** | 2/0.24 | 4/0.52 | 60/5.74 | 30 | 1.5 |
| Tb-SRSO_10_Ar60P60 | **22.16** | 2/0.21 | 4/0.48 | 60/6.02 | 60 | 3.0 |
| Tb-SRSO_11_Ar40P10 | **4.89** | 2/0.22 | 4/0.5 | 40/4.24 | 10 | 0.5 |
| Tb-SRSO_12_Ar40P30 | **12.06** | 2/0.25 | 4/0.56 | 40/4.26 | 30 | 1.5 |
| Tb-SRSO_13_Ar40P60 | **18.80** | 2/0.25 | 4/0.5 | 40/ | 60 | 3.0 |
|  |  |  |  |  |  |  |
| Tb_ORSO_21_Ar60P30 | **4.066** | 2/0.28 | 28/3.04 | 60/6.17 | 30 | 1.5 |
| Tb_ORSO_22_Ar40P30 | **4.375** | 2/0.17 | 28/2.98 | 40/4.20 | 30 | 1.5 |
| Tb_ORSO_23_Ar100P30 | **4.287** | 2/0.32 | 28/3.1 | 100/9.45 | 30 | 1.5 |
| Tb_ORSO_24_Ar40P10 | **0.60** | 2/0.27 | 28/3.07 | 40/4.29 | 10 | 0.5 |
| Tb_ORSO_25_Ar40P60 | **17.43** | 2/0.26 | 28/3.03 | 40/4.26 | 60 | 3.0 |
| Tb_ORSO_26_Ar30P30 | **4.32** | 2/0.26 | 28/3.05 | 20/2.2 | 30 | 1.5 |
| Tb_ORSO_27_Ar10P30 | **4.48** | 2/0.25 | 28/3.01 | 10/1.18 | 30 | 1.5 |
| Tb_ORSO_28_Ar40P40 | **6.12** | 2/0.27 | 28/3.07 | 40/4.2 | 40 | 2.0 |
| Tb_ORSO_29_Ar30P30 |  | 2/0.23 | 28/2.96 | 30/3.27 | 30 | 1.5 |
| Tb_ORSO_30_Ar25P30 | **3.64** | 2/0.28 | 28/3.01 | 25/2.63 | 30 | 1.5 |
| Tb_ORSO_31_Ar15P30 | **3.75** | 2/0.28 | 28/3.03 | 15/1.82 | 30 | 1.5 |
| Tb_ORSO_32_Ar30P40 | **6.09** | 2/0.31 | 28/3.08 | 30/3.33 | 40 | 2.0 |
| Tb_ORSO_33_Ar20P30 | **3.99** | 2/0.28 | 28/3.21 | 20/2.33 | 30 | 1.5 |
| Tb_ORSO_34_Ar40P5 | **0.041** | 2/0.22 | 28/3.02 | 40/4.21 | 5 | 0.3 |
| Tb_ORSO_35_Ar40P20 | **2.034** | 2/0.24 | 28/2.99 | 40/4.22 | 20 | 1.0 |
| Tb_ORSO_36_Ar20P5 | **0.037** | 2/0.24 | 28/2.98 | 20/2.26 | 5 | 0.3 |
| Tb_ORSO_37_Ar20P20 | **1.81** | 2/0.25 | 28/3.01 | 20/2.25 | 20 | 1.0 |
| Tb_ORSO_38_Ar20P10 | **0.55** | 2/0.25 | 28/3.01 | 20/2.26 | 10 | 0.5 |
| Tb_ORSO_39_Ar50P30 | **4.043** | 2/0.26 | 28/2.97 | 50/5.12 | 30 | 1.5 |
| Tb_ORSO_40_Ar20P30 | **3.90** | 2/0.25 | 28/3.06 | 20/2.31 | 30 | 1.5 |
|  |  |  |  |  |  |  |
| Tb_SiO_41_Ar40P10 | **0.59** | 2/0.23 | 15/1.17 | 40/4.19 | 10 | 0.5 |
| Tb_SiO_43_Ar40P20 | **2.23** | 2/0.27 | 15/1.77 | 40/4.27 | 20 | 1.0 |
| Tb_SiO_44_Ar40P40 | **6.96** | 2/0.27 | 15/1.76 | 40/4.29 | 40 | 2.0 |
| Tb_SiO_45_Ar40P60 | **13.48** | 2/0.27 | 15/1.79 | 40/4.28 | 60 | 3.0 |


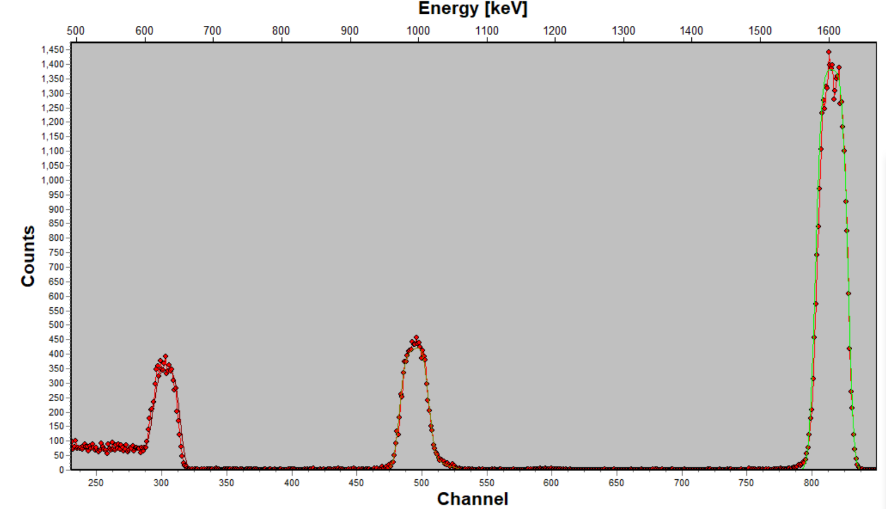
Fig. S2. shows the typical experimental RBS data (red dots) of as-deposited Tb-ORSO thin film grown on graphite substrate and the fit generated by the SIMNRA program (color solid lines).

Considering power as the explanatory variable or input, O value as a parameter, and Tb concentration, refractive index, and thickness as responses or outputs, three piece-wise affine models were learned to capture the relationship between power and each one of the outputs. In these models, partitions are determined based on the O value. Then, linear regression was performed to learn an affine function that relates the input to the corresponding output in each partition. Since samples were fabricated using three different O values, there are three pieces for each model that capture the relationship between power and one of the three outputs. Therefore, in total nine affine functions were learned by performing linear regression. Parameter values for these nine affine functions plus the corresponding Pearson correlation coefficients for each one of these partial models are shown in Table S2.

Table S2. Parameter values for piece-wise affine models learned for different regimes of O value, where power is the input, and Tb concentration, refractive index, and thickness are outputs.

|  | O_2_ = 4 | | | O_2_ = 15 | | | O_2_ = 28 | | |
| --- | --- | --- | --- | --- | --- | --- | --- | --- | --- |
|  | Slope | Intercept | Correlation | Slope | Intercept | Correlation | Slope | Intercept | Correlation |
| Power-Tb | 0.2612±0.02065 | 4.270±0.7789 | 0.9673 | 0.2619±0.01836 | -2.792±0.6413 | 0.9927 | 0.2595±0.02750 | -2.990±0.8190 | 0.916 |
| Power-n | 5.12e-4±0.0014 | 1.853±0.05287 | 0.1096 | 0.003916±2.9662e-4 | 1.420±0.01036 | 0.9915 | 0.004216±4.217e-4 | 1.418±0.01256 | 0.924 |
| Power-d | 4.526±0.39 | -3.786±14.71 | 0.9615 | -0.2060±0.4227 | 81.77±1.476 | -0.9423 | -0.1417±0.1560 | 84.89±4.646 | -0.215 |
|  | O_2_ = 4 | | | O_2_ = 15 | | | O_2_ = 28 | | |
|  | Slope | Intercept | Correlation | Slope | Intercept | Correlation | Slope | Intercept | Correlation |
| Power-Tb | 0.2612±0.02065 | 4.270±0.7789 | 0.9673 | 0.2619±0.01836 | -2.792±0.6413 | 0.9927 | 0.2595±0.02750 | -2.990±0.8190 | 0.916 |
| Power-n | 5.12e-4±0.0014 | 1.853±0.05287 | 0.1096 | 0.003916±2.9662e-4 | 1.420±0.01036 | 0.9915 | 0.004216±4.217e-4 | 1.418±0.01256 | 0.924 |
| Power-d | 4.526±0.39 | -3.786±14.71 | 0.9615 | -0.2060±0.4227 | 81.77±1.476 | -0.9423 | -0.1417±0.1560 | 84.89±4.646 | -0.215 |
|  | O_2_ = 4 | | | O_2_ = 15 | | | O_2_ = 28 | | |
|  | Slope | Intercept | Correlation | Slope | Intercept | Correlation | Slope | Intercept | Correlation |
| Power-Tb | 0.2612±0.02065 | 4.270±0.7789 | 0.9673 | 0.2619±0.01836 | -2.792±0.6413 | 0.9927 | 0.2595±0.02750 | -2.990±0.8190 | 0.916 |
| Power-n | 5.12e-4±0.0014 | 1.853±0.05287 | 0.1096 | 0.003916±2.9662e-4 | 1.420±0.01036 | 0.9915 | 0.004216±4.217e-4 | 1.418±0.01256 | 0.924 |
| Power-d | 4.526±0.39 | -3.786±14.71 | 0.9615 | -0.2060±0.4227 | 81.77±1.476 | -0.9423 | -0.1417±0.1560 | 84.89±4.646 | -0.215 |
|  | O_2_ = 4 | | | O_2_ = 15 | | | O_2_ = 28 | | |
|  | Slope | Intercept | Correlation | Slope | Intercept | Correlation | Slope | Intercept | Correlation |
| Power-Tb | 0.2612±0.02065 | 4.270±0.7789 | 0.9673 | 0.2619±0.01836 | -2.792±0.6413 | 0.9927 | 0.2595±0.02750 | -2.990±0.8190 | 0.916 |
| Power-n | 5.12e-4±0.0014 | 1.853±0.05287 | 0.1096 | 0.003916±2.9662e-4 | 1.420±0.01036 | 0.9915 | 0.004216±4.217e-4 | 1.418±0.01256 | 0.924 |
| Power-d | 4.526±0.39 | -3.786±14.71 | 0.9615 | -0.2060±0.4227 | 81.77±1.476 | -0.9423 | -0.1417±0.1560 | 84.89±4.646 | -0.215 |

Fig. S3. The refractive index and thickness of Tb-ORSO samples fabricated with identical deposition parameters except different range of sputtering powers from 10 to 60 W.


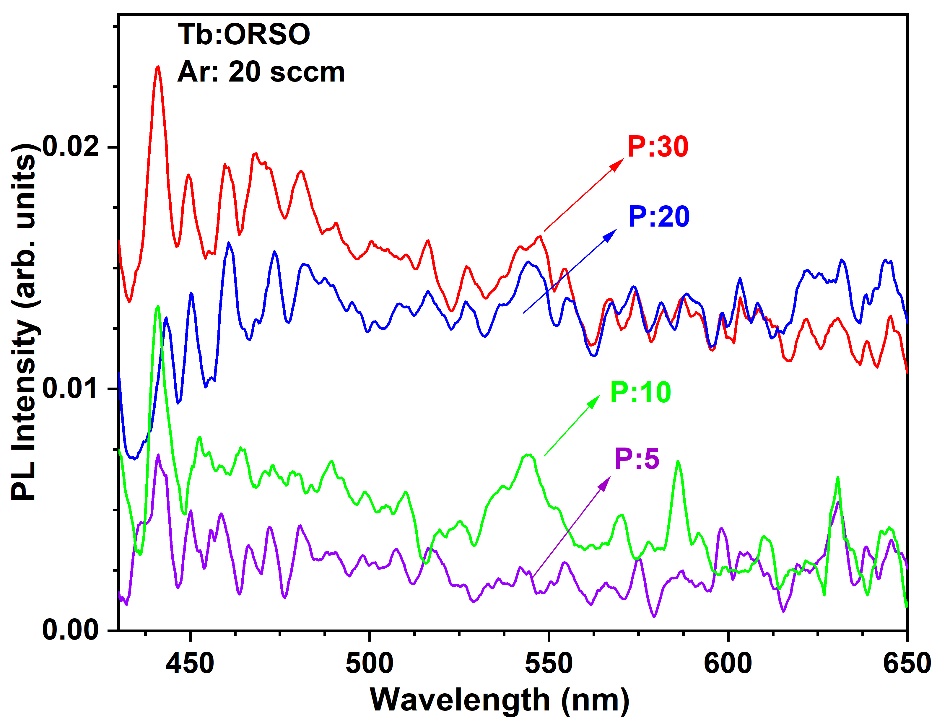


Fig. S4. The PL emission of Tb-ORSO samples fabricated with identical deposition parameters except different sputtering powers ranging from 5 to 60 W.


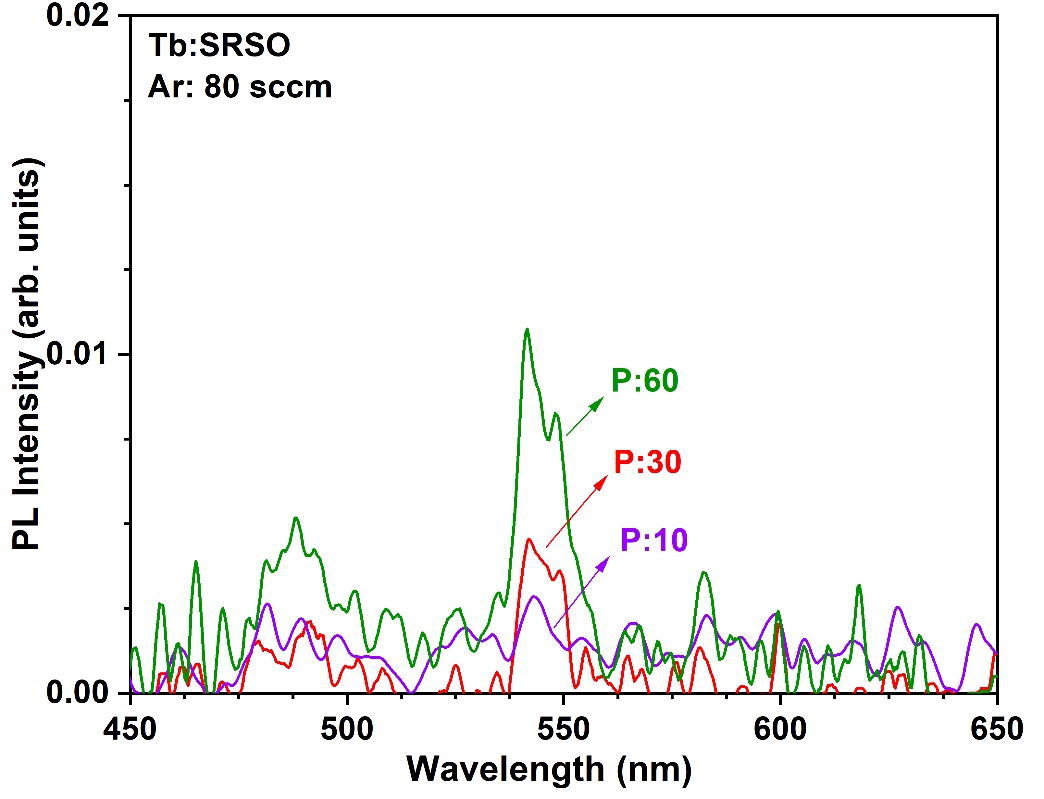


Fig. S5. The increase of power increases the PL intensity of Tb-SRSO samples fabricated using a fixed Ar gas flow rate of 80 sccm.


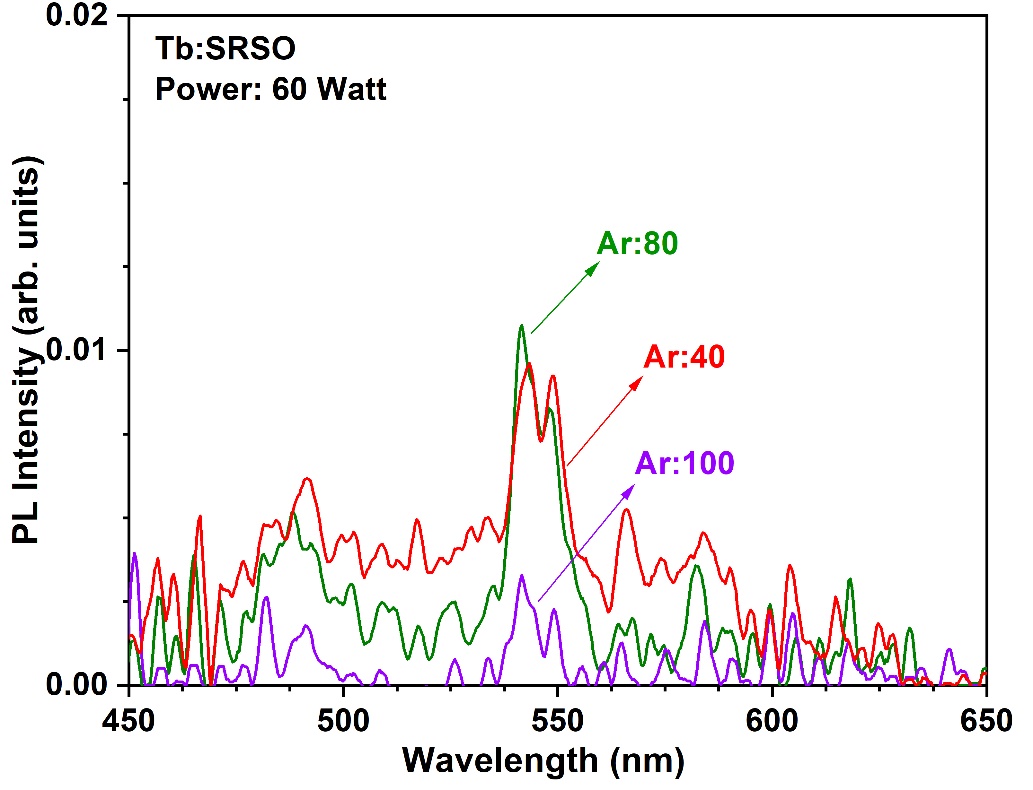


Fig. S6. Dependence of PL emission on the Ar flow rates of SRSO samples fabricated using fixed sputtering power. The increase of Ar gas flow rate to the highest capacity of the mass flow controller (Ar = 100 sccm) decreases the PL emission of Tb-SRSO samples.
